# Hydrogen peroxide initiates oxidative stress and proteomic alterations in meningothelial cells

**DOI:** 10.1038/s41598-022-18548-3

**Published:** 2022-08-25

**Authors:** Xiaorong Xin, Tianxiang Gong, Ying Hong

**Affiliations:** 1 Department of Ophthalmology, Sichuan Provincial People’s Hospital, University of Electronic Science and Technology of China, Chengdu, 610072 Sichuan Province China; 2 Blood Research Laboratory, Chengdu Blood Center, Chengdu, Sichuan China

**Keywords:** Cell biology, Molecular biology, Neuroscience

## Abstract

Meningothelial cells (MECs) are fundamental cells of the sheaths covering the brain and optic nerve, where they build a brain/optic nerve-cerebral spinal fluid (CSF) barrier that prevents the free flow of CSF from the subarachnoid space, but their exact roles and underlying mechanisms remain unclear. Our attempt here was to investigate the influence elicited by hydrogen peroxide (H_2_O_2_) on functional changes of MECs. Our study showed that cell viability of MECs was inhibited after cells were exposed to oxidative agents. Cells subjected to H_2_O_2_ at the concentration of 150 µM for 24 h and 48 h exhibited an elevation of reactive oxygen species (ROS) activity, decrease of total antioxidant capacity (T-AOC) level and reduced mitochondrial membrane potential (ΔΨm) compared with control cells. 95 protein spots with more than twofold difference were detected in two dimensional electrophoresis (2DE) gels through proteomics assay following H_2_O_2_ exposure for 48 h, 10 proteins were identified through TOF/MS analysis. Among the proteomic changes explored, 8 proteins related to energy metabolism, mitochondrial function, structural regulation, and cell cycle control were downregulated. Our study provides key insights that enhance our understanding of the role of MECs in the pathology of brain and optic nerve disorders.

## Introduction

Optic nerve and brain are enveloped with three meninges including dural, arachnoid and pial sheaths. Meningothelial cells (MECs) are predominant cell components covering both the arachnoid and the pia mater of the neuronal tissue and closely contact with cerebral spinal fluid (CSF), which facilitates to build a brain/optic nerve-CSF barrier that prevents the free flow of CSF from the subarachnoid space. MECs are crucial for removal of the active biomolecules through phagocytosis from CSF to maintain the micro-environment balance of the subarachnoid space. Therefore, any pathophysiological changes in MECs might have an impact on the integrity of the brain/optic nerve-CSF barrier^1–5^.

Increasing investigations have demonstrated that oxidative stress is involved in the progression of numerous neurodegenerative diseases such as Alzheimer’s and Parkinson’s diseases, glaucoma, and mitochondrial optic neuropathies^6–9^. The brain and optic nerve are particularly sensitive to oxidative damage due to their high metabolic rate but with a limited capacity of cellular regeneration^9^. Oxidative stress characterized by the overproduction of free radicals plays a pivotal role in these neurodegenerative diseases. Mitochondria have been recognized to be a major site for reactive oxygen species (ROS) production^10^. ROS serve as important signaling molecules, whereas the over-accumulation of ROS in pathological conditions results in oxidative stress^11^. Mitochondrial membrane potential (ΔΨm) is crucial for sustaining the biological activities of the respiratory chain to generate adenosine triphosphate (ATP) and maintaining the normal function of mitochondria, the loss of ΔΨm deprives cells of energy and leads to subsequent death^12,13^. Brain and ocular tissues normally have the potential to balance the mild damage caused by oxidative stress through several intrinsic antioxidant enzymes. However, overproduction of ROS, free radicals, and mitochondrial dysfunction overwhelms the intrinsic antioxidant capacity and results in oxidative stress and progression of pathological damages^7^. We speculate that oxidative stress leads to over-accumulation of ROS, mitochondrial dysfunction in MECs, thereby impairing the protective role of MECs in maintaining the integrity of the brain/optic nerve-CSF barrier, which probably contributes to the brain and optic nerve disorders.

The present study was undertaken to determine the influence of hydrogen peroxide (H_2_O_2_) induced-oxidative stress on cellular functional changes of MECs. Proteomics approach was conducted to detect the protein expression alteration profile upon the oxidative stress. We expected that the results of this study provides new insights into the further understanding of the roles of MECs.

## Results

### Exposure to H_2_O_2_ inhibits cell viability of MECs

**Figure 1:**
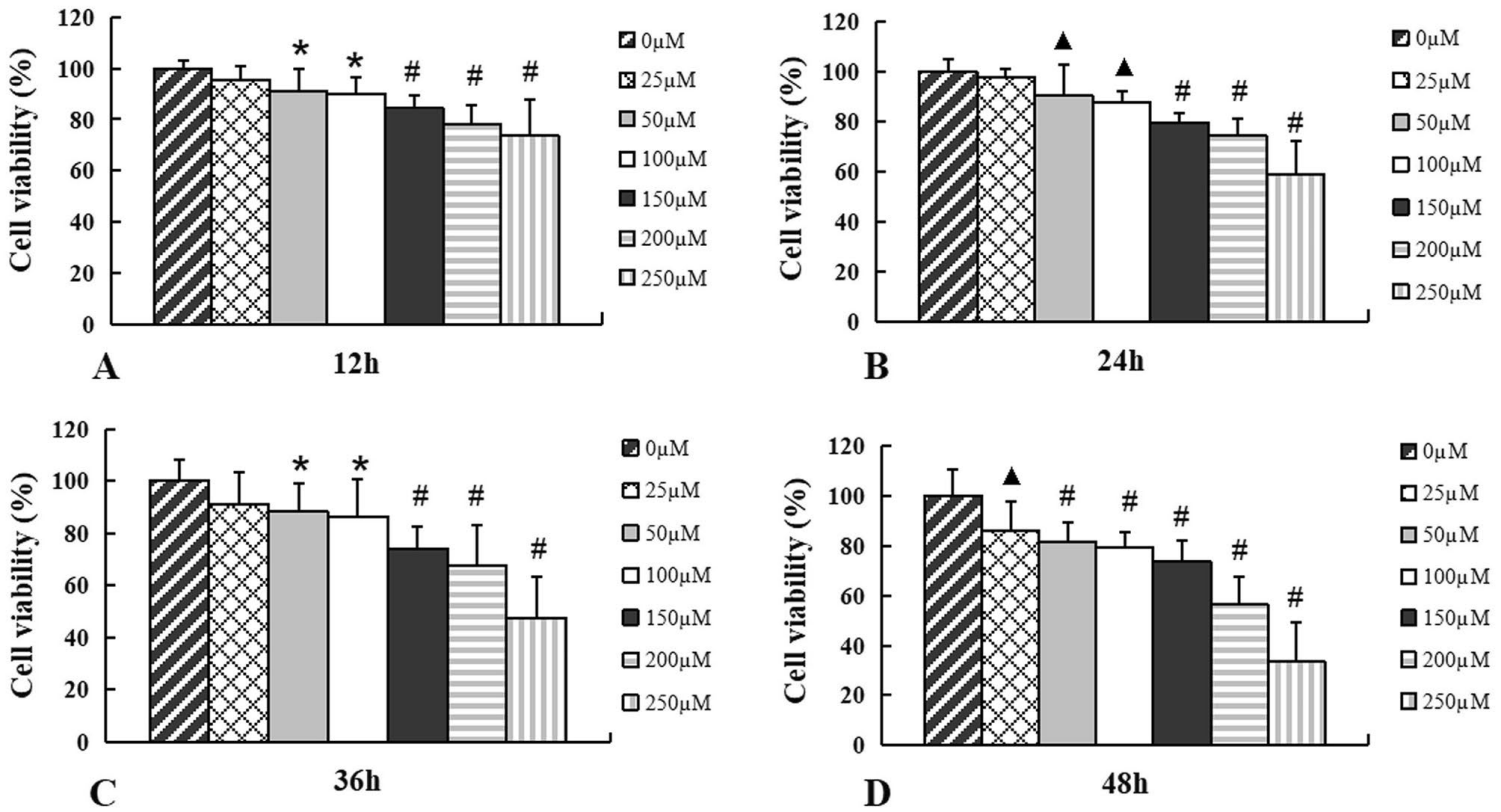
H_2_O_2_ treatment decreases cell viability of MECs. Cell viability of MECs was reduced after exposure to H_2_O_2_ at different concentration for 12 h (**A**), 24 h (**B**), 36 h (**C**), 48 h (**D**) respectively compared with controls. Data are represented as mean ± SD (n = 6),* *p* < 0.05, ^**▲**^*p* < 0.01; ^**#**^*p* < 0.001, control versus H_2_O_2_ treatment groups. MECs, meningothelial cells.

To investigate the effects of H_2_O_2_ on cell viability of MECs, cells were exposed to various concentrations of H_2_O_2_ ranging from 25 to 250 μM for 12 h, 24 h, 36 h, and 48 h respectively. Cell viability in H_2_O_2_-exposed cell groups obviously decreased compared with control cells, which showed an inhibition effect of H_2_O_2_ on cell growth. Significant inhibition effect was presented when cells were incubated with H_2_O_2_ at a concentration of 150 μM at the time of 12 h (*p* < 0.001) (Fig. 1A). Furthermore, a more potent suppressive effect on the MECs cell viability was exhibited with the increase of both the H_2_O_2_ exposure time and treatment concentration (Fig. 1B–D).

### H_2_O_2_ alters the morphology of MECs

**Figure 2:**
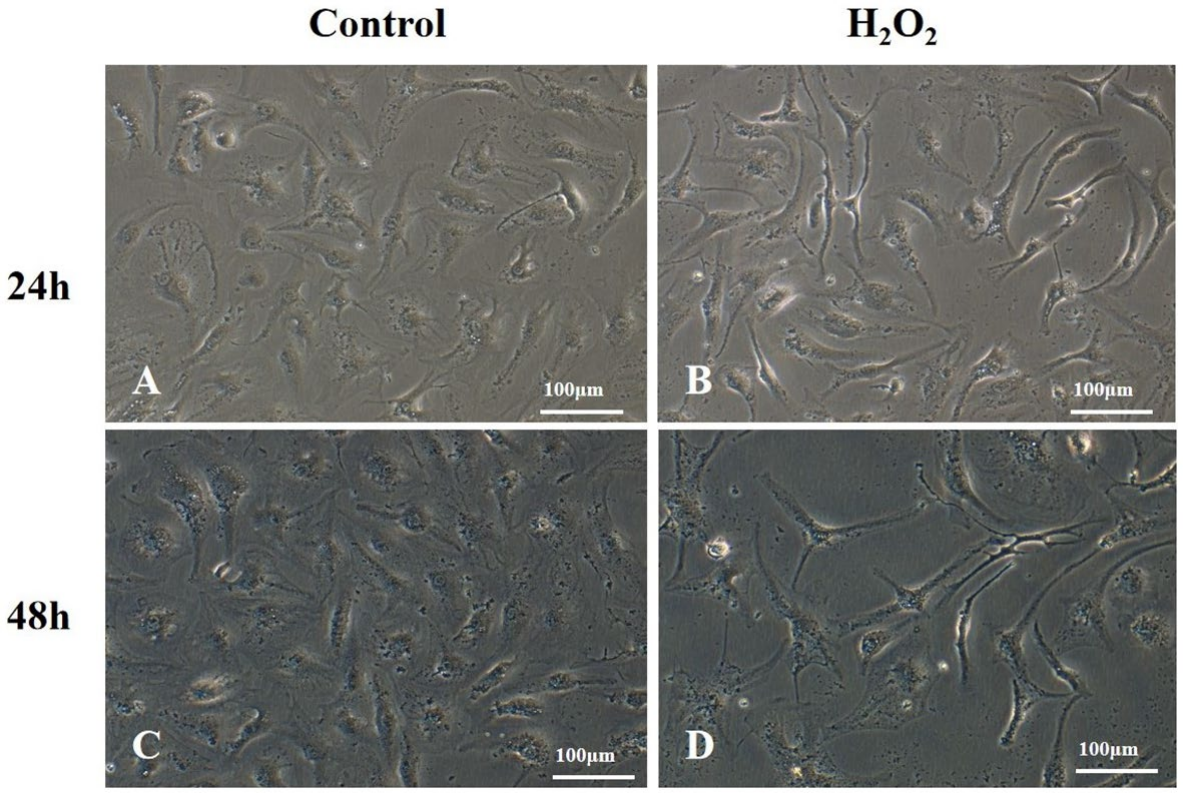
Morphology of MECs under the microscopy. MECs culture images of controls after the incubation for 24 h (**A**) and 48 h (**C**); MECs treated with H_2_O_2_ (150 µM) for 24 h (**B**) and 48 h (**D**). Scale bar: 100 μm. MECs, meningothelial cells.

To identify cellular morphological changes upon the oxidative stress, we observed cells under the inverted microscope. We found that MECs from controls showed increased cellular proliferation and round nuclear after the incubation for 24 h and 48 h (Fig. 2A,C). In contrast, the proliferation of cells treated with H_2_O_2_ at a concentration of 150 μM for the same period was inhibited (Fig. 2B,D). The MECs configuration made a change from round shape into shrunk and elongated ones in response to H_2_O_2_ compared to the control group (Fig. 2B,D).

### H_2_O_2_ suppresses anti-oxidative capability of MECs

**Figure 3:**
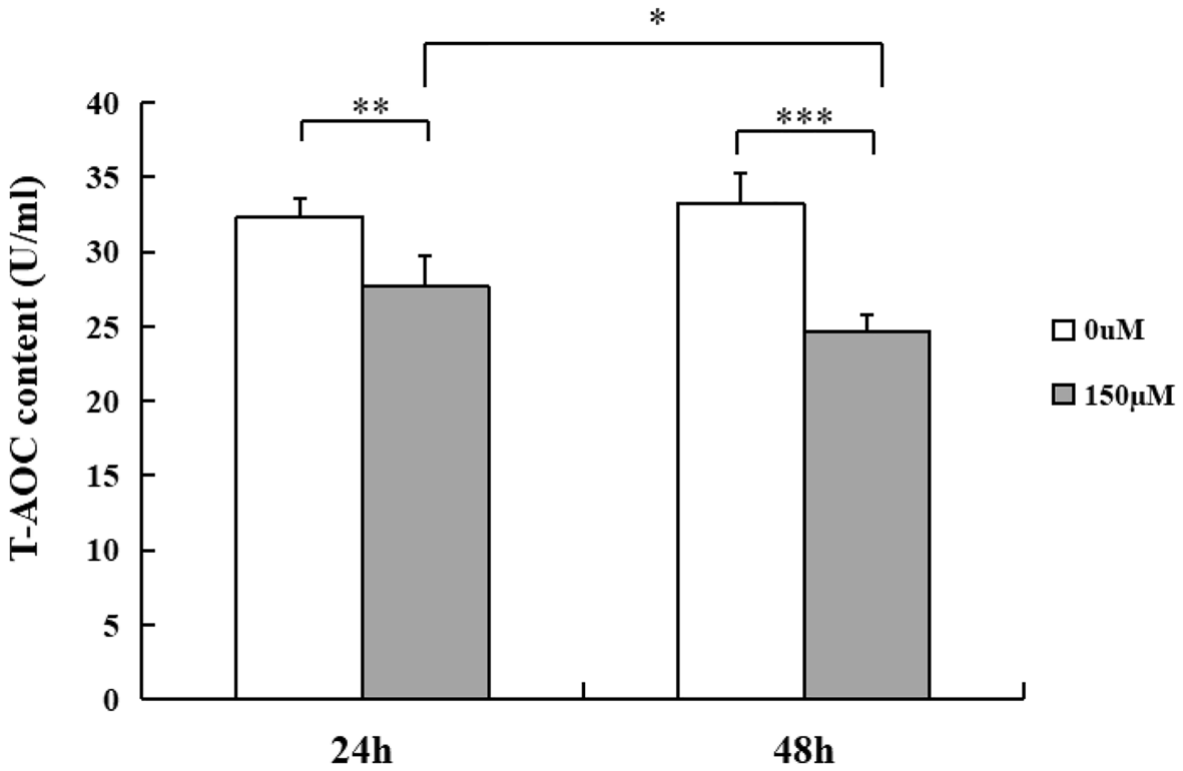
H_2_O_2_ treatment inhibits T-AOC content of MECs. T-AOC content of MECs was decreased after cells were exposed to H_2_O_2_ (150 µM) for 24 h and 48 h respectively compared with untreated cells. Data are represented as mean ± SD (n = 6) *p < 0.05, **p < 0.01, ***p < 0.001. MECs, meningothelial cells; T-AOC, total antioxidant capacity.

In order to assess the oxidative effect of H_2_O_2_ on MECs, we treated cells with H_2_O_2_ at a concentration of 150 µM for 24 h and 48 h respectively. After 24 h exposure, the cells administrated with H_2_O_2_ exhibited a decrease of total antioxidant capacity (T-AOC) production compared with control cells (*p* < 0.01). After 48 h of incubation with H_2_O_2_, a significant reduction of T-AOC content in H_2_O_2_-exposed groups was observed compared with the controls (*p* < 0.001). In comparison with T-AOC level of H_2_O_2_ -treated cell groups for 24 h, the suppressed level of T-AOC was showed in the cell groups with the same treatment for 48 h (*p* < 0.05) (Fig. 3).

### H_2_O_2_ exposure activates ROS in MECs

**Figure 4:**
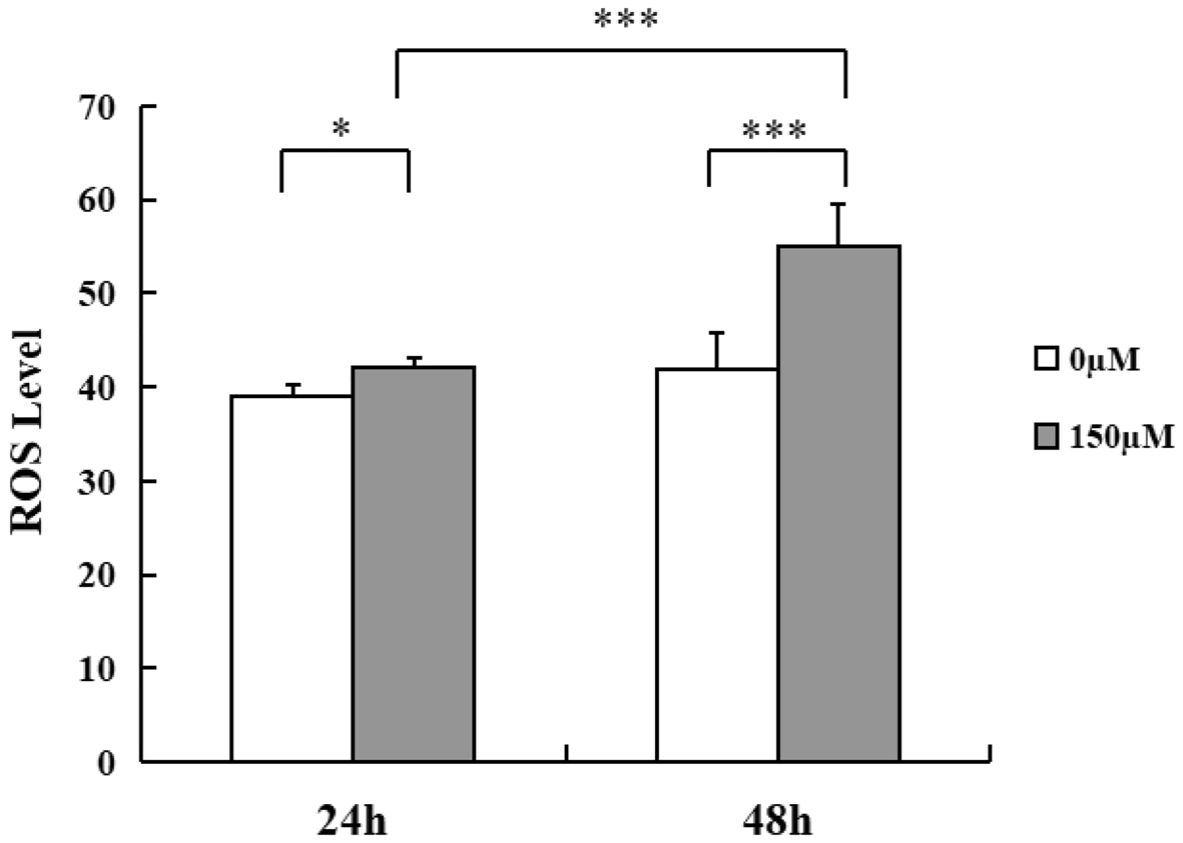
H_2_O_2_ exposure activates intracellular ROS activity of MECs. Quantitative analysis of intracellular ROS in MECs exposure to H_2_O_2_ (150 µM) for 24 h and 48 h respectively. Values are shown as mean ± SD (n = 6). *p < 0.05, ***p < 0.001. MECs, meningothelial cells; ROS, reactive oxygen species.

To further clarify the effect of H_2_O_2_ on ROS activity of MECs, we used 2′, 7′-dichlorodihydrofluorescein diacetate (DCFH-DA) probe to detect intracellular ROS level. As shown in Fig. 4, cells treated with H_2_O_2_ for 24 h exhibited elevated ROS activity compared with the control cells (*p* < 0.05). After 48 h of incubation with H_2_O_2_, ROS level was significantly increased compared with the untreated cells (*p* < 0.001). Increased ROS production was found in the cell groups with H_2_O_2_ treatment for 48 h when compared with cells underwent the same treatment for 24 h (*p* < 0.001), indicating ROS production upon the H_2_O_2_-triggered stress increases in a time-dependent manner. These data suggest that H_2_O_2_ insult results in the excessive ROS generation and accumulation in MECs.

### Effect of H_2_O_2_-induced stress on ΔΨm of MECs

**Figure 5:**
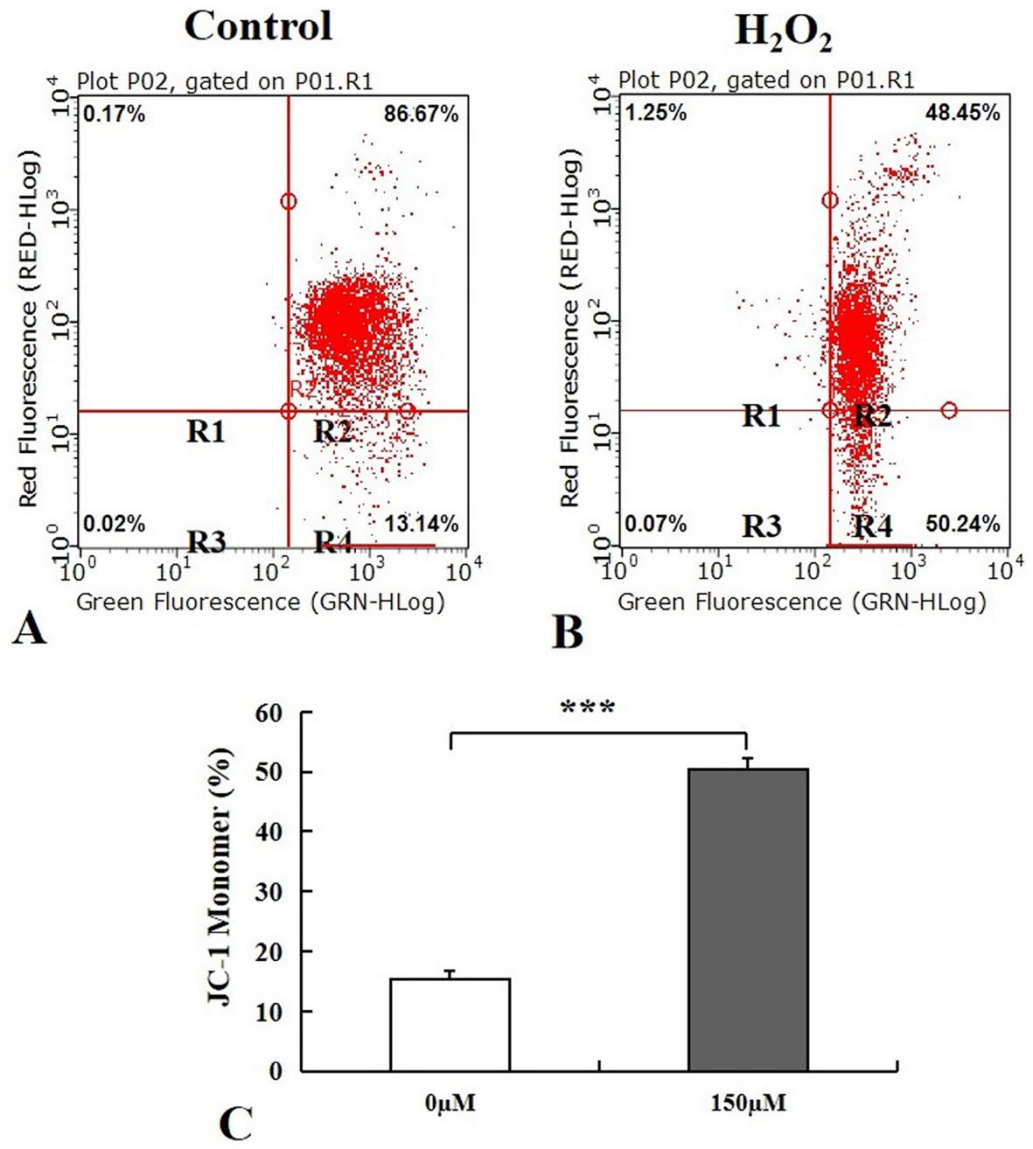
Impact of H_2_O_2_ on mitochondrial membrane potential (ΔΨm) of MECs. Changes in the ΔΨm were analyzed using JC-1 after MECs exposure to H_2_O_2_ (150 µM) for 48 h. H_2_O_2_-exposed cells (**B**) with decreased J-aggregates representing low ΔΨm compared to the controls (**A**); A significant increase in monomer in cells following H_2_O_2_ exposure (**C**). Values are shown as mean ± SD (n = 4). ***p < 0.001. MECs, meningothelial cells; JC-1:5,5′,6,6′ tetrachloro-1,1′3,3′ tetraethylbenzimidazolcarbocyanine iodide.

To assess whether ΔΨm alteration is involved in the mechanism by which intracellular ROS is overproduced after MECs subjects to H_2_O_2_-induced stress, we analyzed ΔΨm using 5,5′,6,6′ tetrachloro-1,1′3,3′ tetraethylbenzimidazolcarbocyanine iodide (JC-1), which is applied for detecting mitochondrial depolarization. MECs were harvested and processed for JC-1 detection with flow cytometry after different treatment. Our results showed that J-aggregates were decreased in H_2_O_2_-exposed cells when compared to the controls (Fig. 5A,B). There was a significant increase in monomer in cell groups with H_2_O_2_ exposure for 48 h (*p* < 0.001) (Fig. 5C), indicating a reduction of mitochondrial ΔΨm in MECs.

### MECs protein profile changes responding to oxidative stress

**Figure 6:**
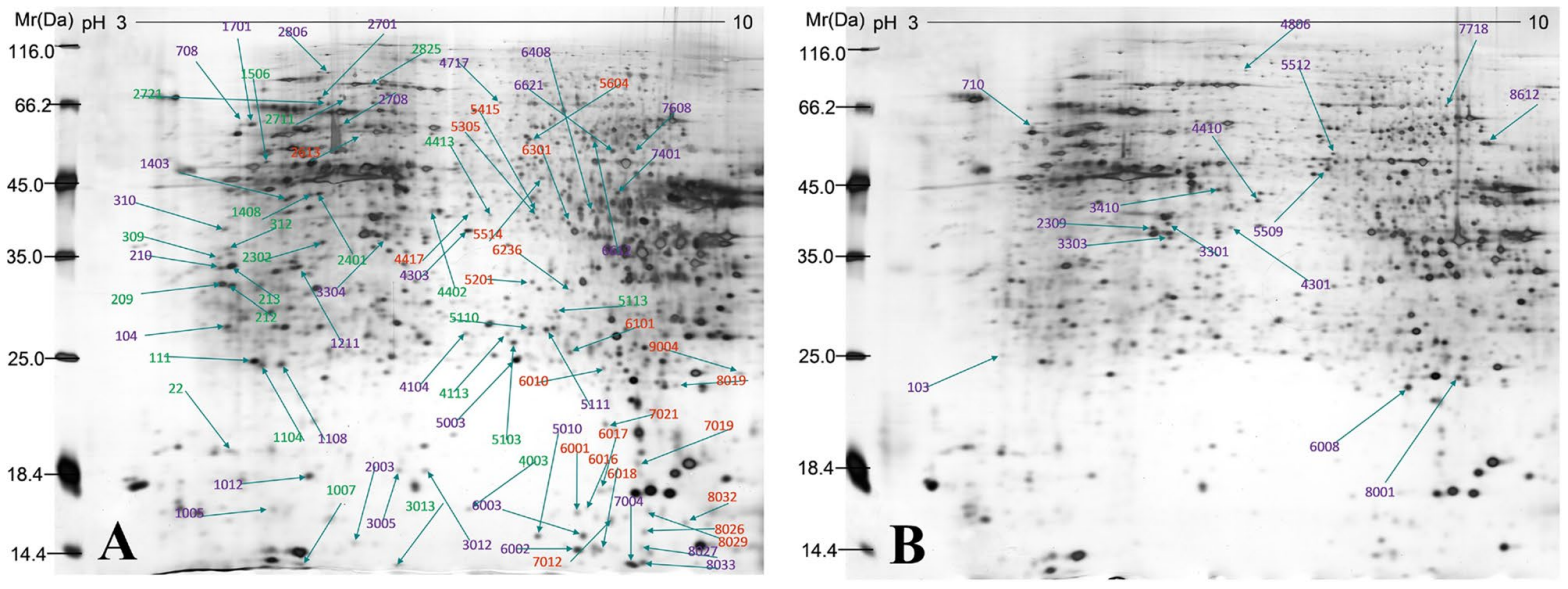
Representative two-dimensional difference gel electrophoresis (2D-DIGE) gel images. Isoelectric focusing (IEF) was performed using 24 cm immobilized pH gradient (IPG) strips covering a linear pH 3–10. (**A**) Shows a 2D gel of total protein from controls; (**B**) shows a 2D gel of total protein from H_2_O_2_**-**treated cells.

To better define the cellular responses to H_2_O_2_, two dimensional electrophoresis (2DE) approach followed by MS analysis was conducted to identify the differentially expressed proteins between controls and H_2_O_2_- treated cells. Statistical analysis of resultant 2-D gels revealed that 95 protein spots were differentially expressed in the two cell groups. Compared to the non-treated cells, 15 proteins were upregulated and 80 were consistently downregulated with fold change ≥ 2-folds (*p* < 0.05). The protein spots appeared in the 2-DE gel images of the control (Fig. 6A) and H_2_O_2_ -treated MECs (Fig. 6B). Proteins with masses varying between 20 and 117 kDa were separated in large format gels along a pH interval of 3–10. In order to obtain the highly significant differentially expressed proteins from our study, the protein differential expression was defined by the criteria of fold change ≥ 3-folds (*p* < 0.01), and a total of 10 significant differentially expressed proteins between the controls and the H_2_O_2_-treated cells were identified. Among these 10 proteins, 8 proteins were downregulated and 2 proteins were upregulated. Table 1 showed Swiss-Prot Accession Numbers, full protein names, theoretical molecular weight (MW), isoelectric point (PI) as well as protein coverage of the identified proteins.

**Table 1:** Differentially expressed proteins in control and H_2_O_2_- exposed cells.

Spot no.	Protein	MW	Protein coverage (%)	PI	Score
1506	Vimentin	49,680	17	5.19	180
2711	Stress-70 protein, mitochondrial	73,920	16	5.87	275
2825	Vigilin	141,995	3	6.43	69
2806	Cytoplasmic dynein intermediate chain 2	71,704	21	5.05	276
3013	Cofilin 1 (Non-muscle)	17,029	37	8.54	70
3303	Guanine nucleotide-binding protein G(I)/G(S)/G(T) subunit beta	38,048	13	5.60	160
5010	Macrophage-capping protein	27,947	20	5.83	148
6018	Nucleoside diphosphate kinase B	22,522	13	7.57	93
8209	Ubiquitin/ISG15-conjugating enzyme E2	17,928	15	7.71	117
8612	ATP synthase subunit alpha, mitochondrial	59,828	7	9.16	202

## Discussion

Emerging evidence demonstrated that MECs is highly active to pathological stress, such as involving in the activation of the immune response by releasing pro-inflammatory cytokines and producing lipocalin-type prostaglandin D synthase to modulate CSF composition under pathological conditions^14–16^. MECs also have a potential role for phagocytosis to remove active molecules or waste products from the subarachnoid space to keep the integrity of brain/ optic nerve-CSF barrier^4^. Impairment of MECs function may be a contributing factor to the disorders of brain and optic nerve.

H_2_O_2_ is a key oxidant to induce free radical and apoptosis, and is therefore widely used in experimental studies as an oxidative stress. H_2_O_2_ has multiple biological effects including mediation of cell proliferation, migration, survival, differentiation, gene expression, and cell death^17,18^. Our study demonstrated that MECs was susceptible to H_2_O_2_-induced oxidative stress. The reduction of MECs viability was exhibited in a dose-dependent and time-dependent manner following exposure to H_2_O_2_. Remarkably, we found that cells configuration changed from round shape into elongated ones upon the microscopic observation of MECs morphology. This finding indicated that H_2_O_2_ -induced oxidative stress causes a cytotoxic impact on MECs and results in the alteration of cell morphology and the decrease of cell viability. Additionally, the decreased T-AOC activity, elevated ROS level and reduced ΔΨm were presented in MECs in response to H_2_O_2_-induced oxidative challenge. Oxidative stress is triggered when the balance between the antioxidant defense system and free radical generation system is disturbed. T-AOC plays a crucial role in the antioxidant defense system by inhibiting ROS and preventing lipid peroxidation through blocking the peroxidation chain^19^. ROS generated by the mitochondrial respiratory chain consists of a number of chemically reactive molecules derived from oxygen, such as superoxide anion and H_2_O_2_. Mitochondrial dysfunction increases ROS production and the oxidative stress ensues if over-accumulation of ROS overwhelms the cellular antioxidant defences. Reduced ΔΨm and increased ROS production have been linked to mitochondrial disorders^20,21^. Our study implies that H_2_O_2_-triggered oxidative stress suppresses total antioxidant capacity, reduces ΔΨm in MECs and thus results in the accumulation of intracellular ROS in MECs. Intracellular ROS overproduction and depolarization of ΔΨm are considered to be important molecular markers for identifying the mitochondrial oxidative stress status. Excessive production of ROS causes a deleterious effect on cellular biomolecules and mitochondria, ultimately leads to the loss of cell viability. Our findings reveal that oxidative stress caused by H_2_O_2_ has a detrimental effect on MECs via intracellular ROS elevation, depolarization of mitochondrial membrane potential, and disruption of the oxidant-antioxidant balance in cells.

In this study, proteomics approach was performed to explore the protein expression profile of MECs to identify proteins involved in the pathogenesis of oxidative stress. A total of 10 stably, significantly dysregulated proteins were detected. Among these identified proteins, vigilin, as a highly conserved protein from yeast to mammals, was downregulated in our study. The diversity of vigilin’s biological roles includes chromosome segregation, translation and tRNA transport, and regulation of mRNA metabolism^22,23^. In our study, down-regulation of vigilin in H_2_O_2_-treated cells might contribute to the inhibition of cellular function to regulate the translational activities of mRNAs under the oxidative stress.

Three actin regulatory-related proteins which were downregulated in our present study include vimentin, macrophage-capping protein (CAPG), and Cofilin-1. Vimentin performs a significant role in supporting and anchoring the position of the cellular organelles by attaching to the nucleus, endoplasmic reticulum and mitochondria. It is also the cytoskeletal component responsible for maintaining cell integrity, cell adhesion and extracellular matrix (ECM) formation^24,25^. The lack of vimentin results in the loss of cell morphology and reduces cell adhesion^26^. In addition, vimentin plays a role in regulating the membrane potential of mitochondria and supporting its high bioenergetic capacity, and vimentin deficiency inhibits ATP synthesis and promotes ROS production under pathological conditions^25,26^. It is highly likely that the decreased vimentin expression upon the oxidative stress in our study may influence its function of maintaining cell shape, integrity of the cytoplasm, stabilizing cytoskeletal interactions and mitochondrial function. CAPG has been identified to be an actin-binding protein and displays a range of activities including regulating cytoplasmic and nuclear structures, modulating cell motility by interacting with the cytoskeleton^27,28^. Cofilin-1 is a nonmuscle isoform of actin regulatory protein that belongs to the cofilin family^29^. Studies of the cofilin family have demonstrated that this molecule regulates a complex series of events such as actin filament turnover, cell cycle progression, migration, cell motility, and formation of cell processes^30–32^. In our study, the dysregulation of CAPG, vimentin, and Cofilin 1 in MECs upon H_2_O_2_ stress could be an important contributing factor to the cell viability loss, cell morphology alteration, and mitochondrial dysfucntion of MECs.

Our study showed that both ubiquitin and nucleoside diphosphate kinases (NDPKs) levels were suppressed in cells subjected to oxidative stress. Ubiquitin was recognized to be a crucial molecule to label intracellular proteins for degradation by a multienzymatic complex^33,34^. E2 enzyme for interferon-stimulated gene-15 (ISG15) is responsible for the attachment of ubiquitin (Ub) to cellular proteins^35^. NDPKs are key enzymes ubiquitously found in all organisms and show remarkable sequence conservation^36^. They catalyze the transfer of terminal phosphates from nucleoside triphosphates (NTP) to nucleoside diphosphates (NDP) to yield their respective nucleoside triphosphates^37^. NDPKB is a DNA-binding protein that recognizes the nuclease-hypersensitive element and maintains the intracellular concentration of NTPs and dNTPs. The downregulation of enzymes including Ubiquitin/ISG15-conjugating enzyme E2 and NDPKB in our study suggests that oxidative stress most likely attenuates their enzymatic role in multiple cellular processes such as cell proliferation, differentiation, and cell development.

Our protemic analysis demonstrated that H_2_O_2_ inhibited the expression of cytoplasmic dynein intermediate chain 2, which is an essential component of the dynein complex and exerts important functions in its cargo recognition, assembly, and regulation^38–40^. The down-regulation of cytoplasmic dynein intermediate chain 2 in H_2_O_2_-exposed MECs indicates a possible mechanism by which the oxidative stress inhibits dynein‐dependent transport, alters dynein motility, affects its major roles in communicating with other protein complexes^41^, and thereby leading to the consequent changes in MECs morphology, adhesion and migration. Dynein involves in the subcellular distribution of vimentin intermediate filaments that are crucial cytoskeletal components contributing to cell shape, motility and organelle positioning^42,43^. The dysfunction of these proteins is consistent with our microscopic observation that MECs cell shape is altered with the proteomic changes. Mitochondrial stress-70 protein is one of heat shock proteins and the most important chaperone found in the mitochondrial matrix^44^. Mitochondrial stress-70 protein plays a crucial role in import and refolding of mitochondrial proteins and is essential for protecting intracellular proteins from heat shock, toxicity, hypoxia and inflammation, ROS accumulation and mitochondrial dysfunction^45–48^. In our experiments, decreased expression of stress-70 protein in the stressed cells may be linked to the increased vulnerability of MECs to the oxidative stress.

Data from our study showed that H_2_O_2_ upregulated guanine nucleotide-binding protein subunit beta (GNBP-B) expression. GNBP-B is involved in several crucial biological processes including modulating various transmembrane signaling systems for cell growth, cellular response to hypoxia, and cell apoptosis^49^. Besides GNBP-B, our proteomic data revealed that ATP synthase subunit alpha was also elevated in cells exposure to H_2_O_2_. Mitochondrial membrane ATP synthase produces ATP from ADP in the presence of a proton gradient across the membrane which is generated by electron transport complexes of the respiratory chain^50,51^. Upregulation of GNBP-B and ATP synthase subunit alpha in our study implies that elevated level of these proteins is associated with an increased demand for energy production by the dysfunctional cells to maintain basic function under the oxidative stress.

MECs, the predominant cellular population at the interface between CSF and neuronal tissue, play a pivotal role in maintaining the function of brain/optic nerve-CSF barrier. In our study, H_2_O_2_-initiated stress decreased cell viability, altered cell morphology, increased intracellular ROS activity and reduced T-AOC level and ΔΨm, indicating that MECs are susceptible to the oxidative-stress attack. Among the proteomic changes explored, proteins related to energy metabolism, mitochondrial function, structural regulation, and cell cycle control were dysregulated in response to H_2_O_2_-triggered stress, which may consequently affect cell viability, oxidant-antioxidant balance, and mitochondrial function of MECs, a possible bio-toxic effect due to the malfunction of brain/optic nerve-CSF barrier and reduced clearance of highly biological active molecules in CSF may ensue. Therefore, any impairment of MECs function probably disturbs the integrity of the brain/optic nerve-CSF barrier, which may involve in the disorder of micro-environment of the subarachnoid space surrounding the brain and optic nerve.

In conclusion, this work provides new insights into the cellular mechanisms associated with the pathophysiology of MECs subject to oxidative stress. These findings may be used as a basis for further understanding the role of MECs in brain and optic nerve disorders.

## Materials and methods

### Cell culture

Human meningothelial cell line (Ben Men cell line) (DSMZ, Germany) was cultured in Dulbecco's modified Eagle's medium (DMEM) (Invitrogen, Carlsbad, CA, USA) supplemented with 10% fetal bovine serum (FBS), penicillin/streptomycin (100 U/mL, 100 µg/mL; Sigma, Germany). Cells were trypsinized after washed with phosphate-buffered saline (PBS) (Sigma, Germany), and supernatant was removed after centrifugation.

### MECs exposure to H_2_O_2_ and cell viability determination

MECs cells were cultured in 96-well plates (Falcon, USA) with cell concentration at 1 × 10^4^ cell/well to adhere overnight. H_2_O_2_ was administered at the concentration ranging from 25 to 250 µM. Cells cultured only with DMEM without H_2_O_2_ were regarded as controls. Cell viability was evaluated by 3-(4,5-dimethylthiazol-2-yl)-5- (3-carboxymethoxyphenyl)-2- (4-sulfophenyl)-2H-tetrazolium (MTS) assay following cells exposure to H_2_O_2_ with different concentration for 12 h, 24 h, 36 h, and 48 h respectively. Cell viability was calculated by *A*_treatment_/*A*_control_ × 100% and *A* represents the absorbance recorded at 490 nm.

### Morphological observation

MECs were seeded in a 96-well plate (1 × 10^4^ cells/well, 100 ul/well) and H_2_O_2_ was added to the culture medium with a concentration at 150 µM. MECs were observed using an inverted microscope (Olympus, Japan) after cells were exposed to H_2_O_2_ for 24 h and 48 h respectively.

### Enzyme-linked immunosorbent assay (ELISA) for the level of T-AOC

After MECs exposure to H_2_O_2_ at the concentration of 150 µM for 24 h and 48 h respectively, the measurement of T-AOC activities was performed according to the manufacturer’s instructions. Briefly, cells were washed twice with ice-cold PBS and then were used for T-AOC level assay (Uscnlife Science & Technology Company, USA). The content of T-AOC was measured at 405 nm using ELISA reader (Nanjing, China).

### Measurement of intracellular ROS level

MECs were placed in 96-well plates (1 × 10^4^ cells/well, 100 ul/well). H_2_O_2_ (150 µM) was applied to the culture medium. Cells were incubated with 50 μL of 20 μM DCFH-DA (Sigma-Aldrich, USA) for 30 min in dark following exposure to H_2_O_2_ for 24 h and 48 h respectively. Fluorescence was read using a multifunctional microplate reader (Thermo, USA) with excitation at 485 nm and emission at 535 nm.

### Detection of mitochondrial membrane potential

Measurements of ΔΨm was preformed through FACS analysis using JC-1 (Calbiochem, USA). Cells were seeded in discs with 10 cm diameter (1 × 10^6^ cells/disc) following exposure to H_2_O_2_ at the concentration of 150 µM for 48 h. MECs were harvested, washed, resuspended in PBS and incubated with JC-1 (0.5 μM) at 37 °C for 15 min, and then washed in PBS and analyzed by with a BD LSR II flow cytometer at an excitation of 488 nm laser and emission at 576 nm.

## Proteomic analysis

### Protein extraction and quantification

Cells were seeded in 10 cm diameter discs (1 × 10^6^ cells/disc). Supernatant was collected and centrifuged after cells were incubated with H_2_O_2_ at a concentration of 150 µM for 48 h, and washed with PBS three times at room temperature. Cell suspensions were thawed and centrifuged at 4 °C for 10 min at 14,000 g. Bradford assay was used for quantification of proteins in suspension.

### Isoelectric focusing

Protein samples mixtured with fresh rehydration buffer to a total of 450 μL were applied to Immobiline™ Drystrip IPG strip (24 cm, pH 3–10 NL, GE healthcare, USA) using a passive rehydration method for 12 h. IEF was performed at 20 °C using Ettan IPGphor (GE healthcare, USA) as follows: 500 V for 1 h, 1,000 V for 1 h, and finally 10,000 V for 1 h.

### Equilibration and sodium dodecyl sulfate polyacrylamide gel electrophoresis (SDS-PAGE)

Following the IEF separation, the IPG strips were equilibrated with an equilibration buffer for 15 min at room temperature, followed by 2.5% iodoacetamide instead of 1% dithiothreitol (DTT) in equilibration buffer for another 15 min. The equilibrated gel strips were removed and rinsed with 12.5% SDS electrophoresis buffer for 10 s. Sealing solution was added to the surface of the SDS-PAGE gel and then moved to the electrophoresis apparatus for electrophoresis at following parameters: temperature at 15℃; running gel for 45 min at 100 V then running gel at 200 V for 8 h (until the bromophenol blue dye fronts reached 0.5 cm from the bottom of the gel). After the electrophoresis, the gel was removed from plates and stained.

### Gel scan and analysis

The gels were run in triplicate for each sample and stained with silver nitrate solution. The stained gel was scanned by the Image Scanner (GE Healthcare, USA) at a resolution of 300 dots per inch. All gel images were processed by three steps: spot detection, volumetric quantification, and matching, using PDquest 8.0 software.

### Digestion

For gel digestion, the gel spot was destained at room temperature for 5 min after the gel spot was washed twice, removed and incubated in 50% acetonitrile (ACN) and 100% ACN. The gels were rehydrated in 2–4 μL trypsin (Promega, Madison, USA) solution for 30 min. Cover solution (25 mmol/L NH_4_HCO_3_) was then added for digestion for 16 h at 37 °C. The supernatants were transferred into another tube, and the gels were extracted once with 50 μL extraction buffer [67% ACN and 5% trifluoroacetic acid (TFA)]. The peptide extracts and the supernatant of the gel spot were combined and then completely dried.

### MS analysis

Samples were re-suspended with 5 μL 0.1% TFA followed by mixing in 1:1 ratio with a matrix consisting of a saturated solution of α-cyano-4-hydroxy-trans-cinnamic acid in 50% ACN, 0.1% TFA. Mixture (1 ul) was spotted on a stainless steel sample target plate. Peptide MS and MS/MS were performed on an ABI 5800 MALDI-TOF/TOF Plus mass spectrometer (Applied Biosystems, Foster City, USA). Data were acquired in a positive MS reflector using a CalMix5 standard to calibrate the instrument (ABI5800 Calibration Mixture).

### Statistical analysis

Independent *t-*test or ANOVA followed by Bonferroni’s post hoc test was applied. Data were expressed as the mean ± standard deviation. For all statistical analyses, the level of significance was set at a probability of 0.05. Statistical analyses were conducted with SPSS 19.0 statistical analysis software (SPSS Inc., Chicago, IL).

## Abbreviations

ACN: Acetonitrile
ATP: Adenosine triphosphate
CAPG: Macrophage-capping protein
CSF: Cerebral spinal fluid
DCFH-DA: 2′, 7′-dichlorodihydrofluorescein diacetate
DTT: Dithiothreitol
DMEM: Dulbecco's modified Eagle's medium
ECM: Extracellular matrix
FBS: Fetal bovine serum
GNBP-B: Guanine nucleotide-binding protein subunit beta
H_2_O_2_: Hydrogen peroxide
IPG: Immobilized pH gradient
ISG15: Interferon-stimulated gene-15
JC-1: 5,5′,6,6′ Tetrachloro-1,1′3,3′ tetraethylbenzimidazolcarbocyanine iodide
MECs: Meningothelial cells
ΔΨm: Mitochondrial membrane potential
MTS: 3-(4, 5-Dimethylthiazol-2-yl)-5-(3-carboxymethoxyphenyl)-2-(4-sulfophenyl)-2H-tetrazolium
MW: Molecular weight
NDPKs: Nucleoside diphosphate kinases
NTP: Nucleoside triphosphates
PBS: Phosphate-buffered saline
PI: Isoelectric point
ROS: Reactive oxygen species
SDS-PAGE: Sodium dodecyl sulfate polyacrylamide gel electrophoresis
T-AOC: Total antioxidant capacity
TFA: Trifluoroacetic acid
2DE: Two dimensional electrophoresis
2D DIGE: Two-dimensional difference gel electrophoresis
